# Battery Ingestion in Children, an Ongoing Challenge: Recent Experience of a Tertiary Center

**DOI:** 10.3389/fped.2022.848092

**Published:** 2022-04-27

**Authors:** Cristina Lorenzo, Sara Azevedo, João Lopes, Ana Fernandes, Helena Loreto, Paula Mourato, Ana Isabel Lopes

**Affiliations:** ^1^Paediatric Gastroenterology Unit, Department of Paediatrics, University Hospital Santa Maria, Centro Hospitalar Universitário de Lisboa Norte, EPE, Lisbon, Portugal; ^2^Gastrenterology Service, University Hospital Santa Maria, Centro Hospitalar Universitário de Lisboa Norte, EPE, Lisbon, Portugal; ^3^Medical School, University of Lisbon, Lisbon, Portugal

**Keywords:** foreign body ingestions, battery ingestion, caustic injury, pediatric endoscopy, button battery

## Abstract

**Introduction:**

Morbidity related to childhood battery ingestions (BI) has increased recently due to the expanding use of larger lithium cells. A prompt endoscopic removal is vital to prevent severe complications in cases of esophageal batteries (EB).

**Materials and Methods:**

A retrospective, descriptive study of admissions for BI requiring endoscopic removal in a tertiary hospital's pediatric emergency department (Jan. 2011/Dec. 2020).

**Results:**

We had 35 cases, with an increasing incidence in the last 6 years; median age, 26 m (8 m-10 years), witnessed ingestion in 86%. On the X-ray: 14 (40%) had an EB, 21 (60%), a gastric battery (GB). Symptoms were present in 57% (100% EB/24% GB), and vomiting was the most frequent (50%). Endoscopy revealed: EB, 13 (37%); GB, 17 (49%); duodenal battery, 1 (3%); no battery, 4 (11%). Median time to removal: EB, 7 h (2 h-21days); GB, 12 h (2 h-3 days). All the patients with EB on the X-ray (14) had severe mucosal injury (Zargar classification): Grade IIIa, 7 (50%); IIIb, 5 (36%); IV, 2 (14%). CT-scan showed perforation in 2 patients (total, 4; 29% of EB). In patients with GB (21), 14 (67%) had mucosal damage; 13 (93%), mild (< Grade III, two esophageal erosions); 1 (7%) IIIa (esophageal ulceration). A statistically significant association between exposure time, younger age or battery size and severity of endoscopic lesions was found in EB location. There were no mortality cases. Acute complications occurred in 57% of EB: infection, 50%; perforation, 29%; pneumomediastinum/stridor, 14%; pneumothorax/subglottic stenosis/hemodynamic instability, 7 vs. 0% GB. Stenosis subsequently developed in 6 (43%) of EB: mild, 4 cases (29%); severe, 2 cases (14%, one resolved after endoscopic dilation; one needed a gastrostomy and esophagocoloplasty).

**Conclusion:**

We verified recent increase in admissions due to battery ingestions and associated complications, despite the availability of an emergency pediatric endoscopy team. The patients with EB had more severe mucosal injury and poorer short/long-term outcomes. Children with GB had milder lesions, although the presence of a GB did not exclude esophageal injury. The availability of actual data from national referral centers will support advocacy efforts among stakeholders, including industry representatives and policy makers, in preventing worldwide button battery injury.

## Introduction

Battery ingestions represent a significant health hazard in pediatric age. Related morbidity and mortality have sharply risen in the last decade (1, 2) due to the expanding use of larger lithium cells in household products (3–6). Although batteries may pass through the gastrointestinal tract uneventfully, they can cause severe or even fatal complications, especially in younger children if lodged in the esophagus (3, 7). Mucosal damage starts as soon as 15 min after the ingestion, and severe damage can occur within 2 h (5, 8); thus, a prompt endoscopic removal is the key to prevent serious complications in cases of esophageal batteries (EB) (3). In patients with gastric batteries (GB), management strategy remains controversial, and a differential approach has been advocated by the latest international guidelines of the North American Society of Pediatric Gastroenterology, Hepatology and Nutrition (8) and of the European Society for Pediatric Gastroenterology, Hepatology, and Nutrition (9).

Highlighting the current relevance of this topic, “Button Battery Ingestion in Children: Never Again” was precisely the theme of the panel discussion hosted by the ESPGHAN during the World Congress of Pediatric Gastroenterology, Hepatology, and Nutrition in 2021 (10).

Since 2012, our Pediatric Department includes an emergency pediatric endoscopy team (consisting of a pediatric or adult gastroenterologist and an endoscopy nurse), which is the sole organized structure, providing endoscopic support on a permanent basis (7 days/week) to the Lisbon Metropolitan Area and to the entire southern area of the country. The present study is a revision of all pediatric cases of battery ingestions, requiring endoscopic removal at our tertiary hospital setting, during a 10-year period. It illustrates the clinical spectrum and outcome, including the severity of short and long-term complications, despite the availability and early intervention of an emergency endoscopy team.

## Materials and Methods

We performed a retrospective, descriptive study of admissions for battery ingestions, requiring endoscopic removal in a Pediatric Emergency Department (ED) from a tertiary hospital in Lisbon (Portugal) over a 10-year period (from January 2011 to December 2020, including the pandemic period).

Right after a patient is admitted at the Pediatric ED for battery ingestion (or before referral from another hospital, if applicable), the emergency endoscopy team and, subsequently, the emergency anesthesiology team are informed in order to perform an endoscopic removal of the battery without delay in the most coordinated way.

Endoscopic removal in cases of EB is performed emergently (ideally in the first 2 h after admission) regardless of time of ingestion, symptoms or fasting compliance. Batteries located beyond the esophagus are removed as soon as possible in symptomatic children, in cases of ingestion of more than one battery and in the case of co-ingestion with a magnet. We also performed endoscopic removal at the earliest time if a battery remains in the same position (gastric or duodenal) after 24 h or in cases of unknown time of ingestion. Nevertheless, the ultimate decision about timing of battery removal is up to the endoscopy team, considering each case's individual circumstances.

### Sample Selection

Children and adolescents from 0 to 17 years and 364 days old admitted to the ED in the above-mentioned period due to battery ingestion, requiring endoscopic removal, were included.

We excluded cases of ingestion of other foreign bodies, the patients who needed endoscopic removal of batteries in non-gastrointestinal locations (e.g., respiratory tract) or those who did not require endoscopic removal (the asymptomatic patients with a single battery located beyond the esophagus, with progression observed on serial radiographs).

### Data Collection

Study design was retrospective, including prospectively collected data–patients' electronic clinical files and endoscopy records. Information about socio-demographic data (including age, gender, chronic disease), battery ingestion's details (date and place of ingestion, if it was witnessed or unwitnessed, type and number of swallowed batteries, specific symptoms and reason for attending the ED), and in-hospital management (physical examination, location on the X-ray, results of other complementary tests, and time from ingestion to endoscopic removal) was obtained.

Regarding endoscopic findings, we documented the position and battery type, the severity of mucosal injury (in accordance with Zargar's grading classification of caustic lesions) (11), if endoscopic removal was successful and the presence and severity of acute complications.

Additionally, complications during hospital stay, need for additional procedures, and length of stay were analyzed. After discharge, data on long-term complications and duration of follow-up were gathered.

### Data Analysis

Descriptive analysis of all variables was performed using Microsoft Excel 2013^®^ and IBM-SPSS 25.0^®^. A multivariate analysis considering the classical risk factors associated with greater severity of endoscopic lesions was performed (esophageal location; age, <5 years; battery size; time from ingestion to removal).

## Results

During the study period, 35 patients were admitted to our Pediatric ED due to battery ingestion, requiring endoscopic removal, mostly referred from other hospitals (*n* = 31, 89%).

**Figure 1:**
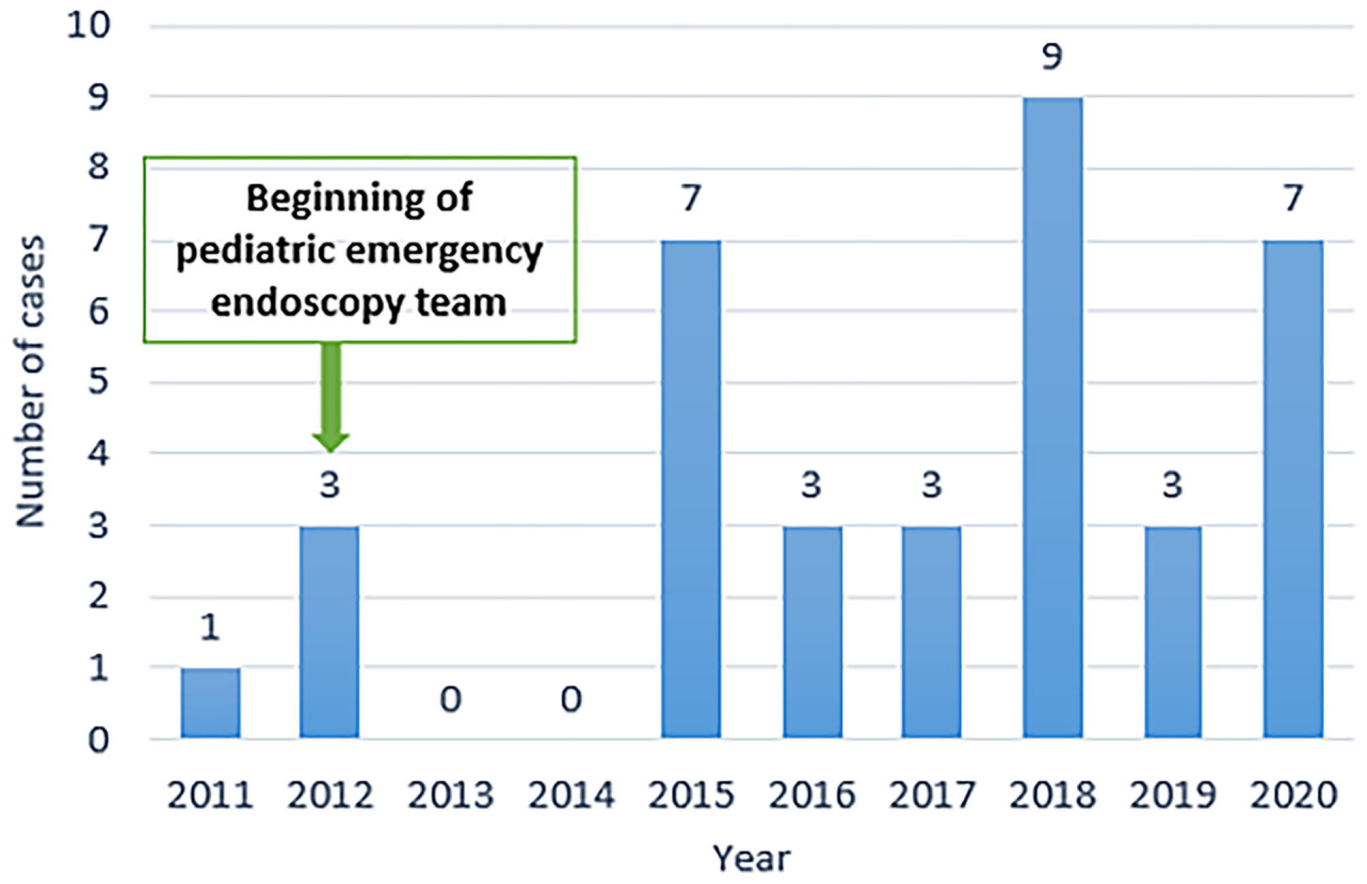
Number of battery ingestion episodes per year.

A recognizably growing incidence in the last 6 years was noticed, with a peak incidence of 9 cases in 2018 (Figure 1).

### Demographic Characteristics

Pediatric female patients accounted for 57% (*n* = 20) of all battery ingestions. Median age was 26 months (minimum, 8 months–maximum, 10 years) and was similar in patients with EB and GB, with a median age in the first group of 20 months (minimum, 8 months–maximum, 10 years) and in the second one, of 29 months (minimum, 15 months–maximum, 9 years).

Most patients were previously healthy (*n* = 28, 80%); 7 patients (20%) had a chronic disease: respiratory disease in 5 (recurrent wheezing/asthma, 4; bronchopulmonary dysplasia, 1) and a developmental disorder in 2 (autism spectrum disorder in a 10-year-old patient, the oldest one in our sample; genetic disorder in another one).

### Circumstances Regarding Ingestion

Battery ingestion suspicion was the reason for attending the ED in 30 cases (86%); in 26 cases (74%), ingestion was directly witnessed by the caregivers, while, in 4 cases (11%), children immediately reported the ingestion. In 5 cases (14%), battery ingestion was not evenly suspected. In 25 cases, ingestion occurred at home. In the remaining cases (*n* = 10), information was not available.

In the 5 cases with unknown history of ingestion (all of them had EB), the patients came to the ED due to the presence of non-specific symptoms: fever (5/5, 100%), vomiting and prostration (2/5, 40%), refusal to feed (2/5, 40%), and neck pain and stiffness (1/5, 20%).

### Battery Location on the X-Ray and Clinical Symptoms

All 35 patients performed a two-view neck, chest, and abdominal radiograph. In 14/35 patients (40%), an opaque foreign body with a “halo” sign consistent with a battery was identified in the esophagus: 8 (57% of EB ingestions) at the cervical level, 3 (21% of EB ingestions) at the thoracic level, as well as 3 cases at the abdominal level. The battery was in the stomach in 21/35 cases (60%).

Symptoms were present in 20/35 patients (57%); all patients with EB (14/14) were symptomatic; on the contrary, only 5/21 patients with GB (24%) had symptoms. More than one symptom was present in 12 cases (34%).

The most frequently associated symptom was vomiting, present in 10/20 cases (50% of symptomatic patients, 8 with EB, 2 with GB). Other symptoms were sialorrhea in 6 (30%, 4 with EB and 2 with GB), fever in 4 (20%, all with EB), refusal to feed and prostration, with 3 cases each (15%, all of them with EB). In symptomatic patients with EB (14/14), most frequently found symptoms were vomiting (*n* = 8, 57%), sialorrhea (*n* = 6, 43%), fever (*n* = 4, 29%), refusal to feed (*n* = 3, 21%), and prostration (*n* = 3, 21%). In symptomatic children with GB (5/21), main symptoms were vomiting (*n* = 2, 40%, one patient had a Grade II aesopagheal injury, and one had no endoscopic lesions), sialorrhea (*n* = 2, 40%, one was the same patient with Grade II aesophageal injury; another one had Grade I gastric lesion), abdominal pain (*n* = 1, 20%, with Grade II aesophageal and gastric injury), melenas (*n* = 1, 20%, with Grade I gastric injury).

Physical examination performed at the ED showed pathologic findings in 7/35 patients (20%), all with EB (50% of the patients with EB).

### Endoscopic Removal

All the patients (35) performed an upper endoscopy. A battery was identified in 31/35 cases (89%). Endoscopy confirmed the esophageal location of the battery in 13/14 patients with EB identified in X-ray (93%). In the other case, the patient vomited and expelled the EB immediately before endoscopy. Considering GB, endoscopy confirmed the gastric location of the battery in 17/21 GB identified in the X-ray (81%). In 3/21 cases (14%), the foreign body was not detected in the endoscopy due to the progression along the gastrointestinal tract, beyond the reach of the endoscope; and, in one patient, the battery was found in the 2^nd^ portion of the duodenum.

Regarding EB, the battery was located respectively, at the cervical, thoracic, and abdominal levels in 8, 3, and 2 cases.

#### Battery Type and Number

Ingestion of a single battery occurred in 30/31 patients in whom a battery was identified during the endoscopy (97%); in one case (3%), two batteries were identified. All cells (31/31) were lithium batteries, and 30/31 (97%) were button batteries. One was an AA cylindrical battery (3%) and was located in the distal part of the esophagus.

Information about size in cases of esophageal batteries detected on the endoscopy was available in 8/13 (62%); in 2 (25%), the diameter was < 15 mm; in 5, (63%) the diameter was ≥ 15 mm (in 2 cases, the diameter was 20 mm); and in one case (12%) was cylindrical. In cases of GB endoscopically detected and with known size (13/18), the diameter was < 15 mm in 5/13 (38%), ≥ 15 mm in 8/13 (62%, 3 were 20-mm batteries, and one was > 20 mm).

Data on battery condition (whether it was new or used), battery code or a source was unavailable.

#### Battery Removal

Endoscopy was effective in removing the battery in 29/31 cases in which the battery was identified (94%). In 2/31 patients (6%), who had a battery in the cervical portion of the esophagus, removal through rigid esophagoscopy was performed successfully after conventional endoscopy failure. There were no cases requiring surgical removal.

#### Time From Ingestion to Removal

Regarding EB, although endoscopy was performed shortly after hospital admission in all cases (14), median time from ingestion to battery removal was 7 h (minimum, 2 h–maximum, 21 days). Removal of EB was performed in the first 2–6 h in 3/14 cases (21%), between 6 and 24 h in 6/14 (43%) and > 24 h in 5/14 (36%). Late removal (≥ 6 h after the ingestion) was performed in the majority of the patients (11/14, 79%) due to a delay in referral from other hospitals in 6/14 (55%, one of them also due to misdiagnosis–the battery was mistaken for an artifact in the 1^st^ visit to the ED), and because of unknown history of battery ingestion in 5/14 (45%).

In GB, median time from ingestion to removal was 12 h (minimum, 2 h–maximum, 3 days).

### Endoscopic Findings

**Figure 2:**
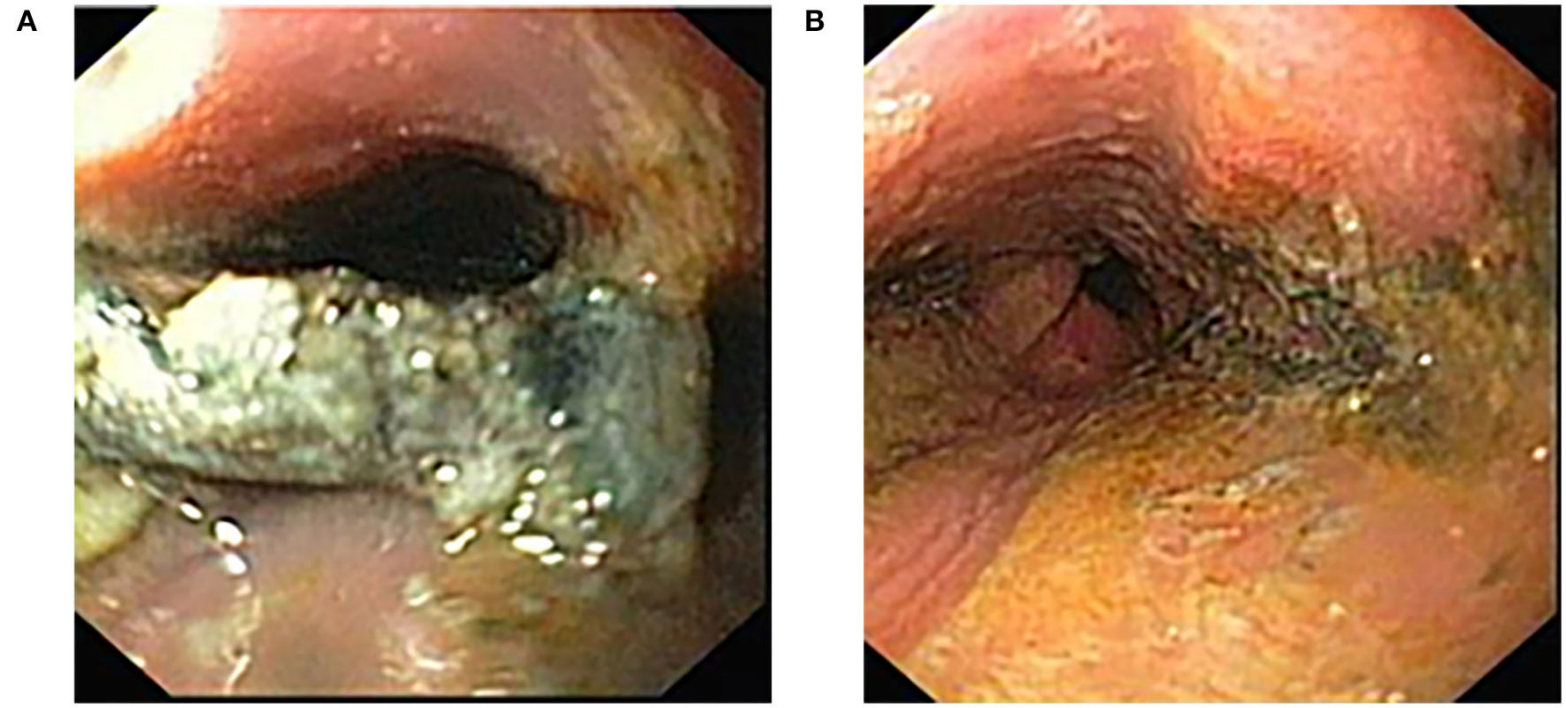
**(A)** Endoscopic image of a battery in distal esophagus of a 6-year-old child with extravasation of its content and covered with necrotic tissue. **(B)** Endoscopy showing circumferential wall necrosis of the distal esophagus immediately following battery removal.

All the patients with an EB on the X-ray (14, including the patient that had vomited and expelled the EB before endoscopy) presented with different grades of esophageal caustic injury according to Zargar classification: Grade IIIa (focal deep gray or brownish-black ulcers) in 7 (50%), Grade IIIb (extensive deep gray or brownish-black ulcers) in 5 (36%, including the patient with a cylindrical battery) and Grade IV in 2 (perforation, 14%). Nevertheless, the CT-scan performed after endoscopy revealed perforation in 2 additional patients–one who was endoscopically classified as Grade IIIa and another one classified as Grade IIIb (total: 4 perforations, 29% of EB) (Figures 2A,B).

In patients with a GB on the X-ray (*n* = 21), mucosal injury was detected in 14/21 (67%)–gastric injury in 11, esophageal in one, both esophageal and gastric lesions in 2. In 13/14 (93%), lesions were minor (< Grade III injury). Only one case (7%) had a major lesion (Grade III ulceration in esophageal-gastric transition and Grade II agastric ulceration). The lesions were classified as Grade I in 6/14 (43%; these patients had only gastric injuries), Grade IIa in 7/14 (50%; in 5 patients, only gastric lesions were identified; in one patient, an isolate esophageal erosion was detected; and the other one had both esophageal and gastric erosions) and Grade IIIa in 1/14 (7%, a patient who had esophageal deep ulceration and superficial gastric ulceration). In 7/21 patients (33%), there were no mucosal injuries identified (Grade 0).

In patients with a GB on the X-ray and in whom no battery was identified on the endoscopy (3/21), mucosa was normal in two and had a Grade IIa gastric injury in one.

### Relationship Between Esophageal Location, Time Until Removal, Younger Age, Battery Size, and Endoscopic Lesions

In EB, mean time from ingestion to battery removal in patients with Grade IIIa mucosal injury was 6 h (minimum, 2 h–maximum, 21 days) in cases of Grade IIIb was 7.5 h (minimum, 2.5 h–maximum, 3 days) and in Grade IV (perforation) was 7 days. Nevertheless, if we considered all 4 patients with perforation (2 detected on the endoscopy and 2 on the CT-scan), median time until removal was 88 h (minimum, 2 h–maximum, 7 days). No statistically significant relationship between time from ingestion to removal and severity of lesions was identified (*p* = 0.18).

Even though patients with perforation were younger (median age between 4 patients with perforation of 13 months *vs*. 26 months in all the patients with an EB), this association was not statistically significant (*p* = 0.12). This could be explained by the fact that almost 75% of our samples included children younger than 5 years. Association between battery size and higher risk of esophageal impaction (*p* = 0.24) or severity of mucosal lesions (*p* = 0.2) was not statistically significant. The only statistically significant association was observed between esophageal location and severity of mucosal lesions (*p* = 0.02, particularly in batteries located in cervical esophagus–*p* = 0.015).

### Complementary Exams

Chest CT-scan was performed in 9 patients (26%); all of them with EB. It was performed before endoscopy in 4 cases with prolonged ingestion, revealing esophageal wall thickening (*n* = 1), perforation with pneumomediastinum (*n* = 1) or showed no alterations (*n* = 2). In 5 cases of EB, the CT-scan was performed after endoscopy due to detection of major endoscopic lesions to exclude potential complications. Perforation was confirmed in 3 cases and esophageal wall thickening in one case.

### Short-Term Follow-Up

#### Need for Hospitalization

All the patients with EB on the X-ray (*n* = 14) were admitted to the Pediatric Gastroenterology or to the Surgery ward; 4 patients (29%) were admitted to the Pediatric Intensive Care Unit (PICU) after endoscopy due to clinical instability. Only 2/21 patients with a GB on the X-ray (10%) were admitted to the Pediatric Gastroenterology ward after endoscopic recovery, none of them in the PICU.

#### Acute Complications

More than half of the patients with EB (8/14, 57%) presented with acute complications. Infection was the most frequent complication, present in 7 patients (89% of patients with lesions, 50% of patients with EB); lower respiratory tract infection in 4, mediastinitis in 3 (with sepsis in 2). Perforation was present in 4 patients (50% of patients with acute complications, 29% of EB), and, in three cases, it was associated with mediastinitis.

One case of EB had major complications with a mean time from ingestion to removal of 8 h; this patient had been previously submitted to an unsuccessful endoscopy (within < 3 h from the ingestion) in a secondary hospital and subsequently transferred to our center, where the EB was successfully endoscopically removed. Perforation and mediastinitis were immediately identified evolving to an extensive and recurrent pneumomediastinum and pneumothorax with a need of a chest drain placement, recurrent stridor, subglottic stenosis, and respiratory distress syndrome (RDS) (requiring prolonged invasive and non-invasive mechanical ventilation) and septic shock with hemodynamic instability needing aminergic support.

Recurrent stridor, pneumomediastinum, and RDS were also observed in 2 other patients (25% of patients with EB and complications, 14% of all EB). Other complications included extensive pneumothorax (*n* = 1), subglottic stenosis (*n* = 1), and hemodynamic instability (*n* = 1)–all of them identified in the above-mentioned patient; 13% of the patients with EB and complications, 7% of all EB). Main characteristics and clinical evolution of the patients with EB and complications (7/14) are displayed in Table 1. All the patients with unknown history of battery ingestion (5/14) had acute complications, but none of the patients with a GB on the X-ray (*n* = 21).

**Table 1:** Esophageal battery ingestion-related complications.

**Sex**	**Age (months)**	**Witnessed ingestion**	**Location**	**Symptoms**	**Battery type and size**	**Time until removal**	Mucosal lesions^*^	**Acute complications**	**Procedures**	**Length of stay**	**Chronic complications**	**Follow-up**
F	13 Mo	Not	Upper third	Fever, sialorrhea, refusal to eat	BB Unknown	7 days	Grade IV	Mediastinitis and sepsis Perforation	Enteral nutrition Antibiotics PPI	1 month	Mild stenosis resolved after 3 months	8 months
F	26 Mo	Yes	Upper third	Vomiting	BB Unknown	17 h	Grade IIIb	Respiratory infection	Parenteral nutrition Antibiotics CVC, PPI	21 days	Mild stenosis resolved after 3 months	4 months
F	20 Mo	Yes	Upper third	Vomiting, sialorrhea, irritability	BB 15 mm	8 h Upper endoscopy Performed twice	Grade IIIb/ Perforation on CT-scan	Mediastinitis, sepsis, perforation, pneumothorax, pneumomediastinum, stridor, subglottic stenosis, RDS, hemodynamic instability	Parenteral + enteral nutrition, gastrostomy, CVC, antibiotics, PPI, invasive/non-invasive ventilation, aminergic support, corticoid therapy, thoracic tube, cervicostomy	80 days (also, in PICU)	Severe stenosis (10 cm length), not resolved after two endoscopic dilations. Gastrostomy feeding. Esophagocoloplasty. Poor weight gain	Maintain follow-up (after 3 years)
F	34 Mo	Not	Upper third	Fever, vomiting prostration	BB 15 mm	28 h	Grade IIIa	Respiratory infection	Enteral nutrition Antibiotics	26 days (also in PICU)	Not	None
M	8 Mo	Not	Middle third	Fever, neck pain and stiffness	2 BB 10/13 mm	21 days	Grade IIIa	Respiratory infection	Enteral + parenteral nutrition Antibiotics CVC	21 days (also in PICU)	Mild stenosis, resolved after 2 months Choking episodes	Maintain follow-up after 2.5 years
M	19 Mo	Not	Upper third	Fever, vomiting, prostration	BB Unknown	3 days	Grade IIIb	Respiratory infection	Enteral + parenteral nutrition Antibiotics PPI, CVC	1 month	Mild stenosis resolved after 3 months	3 months
M	13 Mo	Yes	Upper third	Cough, RDS	BB 15 mm	2 h	Grade IIIb Perforation on CT-scan	Perforation, RDS, stridor, pneumomediastinum	Enteral nutrition Antibiotics, PPI Invasive ventilation	11 days (also in PICU)	Not	2 months
M	15 Mo	Not	Distal third	Refusal to eat	BB 20 mm	7 days	Grade IV	Mediastinitis Perforation	Enteral, parenteral nutrition Antibiotics, PPI, CVC	40 days	Severe stenosis, resolved after one endoscopic dilation, choking episodes	10 years

*BB, button battery; CT, computed tomography; CVC, central venous catheter; F, female; M, male; Mo, months; PICU, pediatric intensive care unit; PPI, proton pump inhibitors; RDS, respiratory distress syndrome*.

* *According to Zargar classification*.

#### Additional Procedures/Therapeutic Interventions

All the patients with EB (*n* = 14) needed additional therapeutic interventions. Proton pump inhibitor (PPI)/sucralfate was initiated in 14 (100%), antibiotics in 10 (71%), and central venous catheter placement in 4 (29%). Feeding support was required in 11/14 patients (79%): enteral nutrition through a nasogastric or nasojejunal tube in 5, parenteral nutrition in one, and both enteral and parenteral nutrition in 5 patients. In two patients (14%, both with esophageal perforation), invasive and non-invasive ventilation was needed due to severe RDS. Systemic corticoid therapy and serial fibrolaryngoscopies were also required in two patients due to recurrent stridor. The above-mentioned patient with a more complicated clinical outcome was the only one who needed a distal esophageal exclusion and a gastrostomy, additionally to a chest drain placement (extensive pneumothorax) and aminergic support (hemodynamic instability). There were no mortality cases, neither other complications such as tracheoesophageal fistula (TEF), aortoesophageal fistula (AEF), or vocal cord paralysis. In patients with GB, there was a need for extra therapeutic interventions in 10/21 patients (48%); in 9/10 (90%, 43% of all the patients with GB) only PPI/sucralfate administration was needed. In one patient (10%, 5% of all of GB) large-spectrum antibiotics and enteral nutrition through a nasojejunal tube were needed.

#### Length of Stay and Median Follow-Up Time

Median length of stay in EB was 21 days (minimum, 2 days–maximum, 80 days); 13/14 patients (93%) were discharged on oral feeding, and one patient (7%) needed a gastrostomy. After discharge, 7/14 patients (50%) were referred to the Pediatric Gastroenterology day hospital or consultation, 2/14 (14%) to Pediatric Surgery consultation, 4/14 (29%) to both consultations, and one patient (7%) was home discharged. Concerning GB, median length of stay was 9 h (minimum, 3 h–maximum, 4 days). All the patients (*n* = 21) were discharged on oral feeds. After discharge, 4/21 patients (19%) were transferred to secondary care centers; 2/21 (10%) were referred to the Pediatric Gastroenterology consultation, and 15/21 (71%) were home discharged.

In cases of EB, median follow-up time of patients who were referred to the Pediatric Gastroenterology or Surgery consultation (*n* = 13) was 2 months (minimum, 1 month–maximum, 10 years). Only 4/13 patients (31%) currently maintain follow-up, respectively: (a) 10 months after the ingestion (a 6-year-old girl with developmental delay and swallowing difficulties), (b) 2 years after (due to frequent choking episodes, with a normal endoscopy, without stenosis); (c) 3 years after (the patient with the most complicated evolution), and (d) 10 years after (a patient with irregular follow-up, with chocking episodes and epigastralgias, but normal endoscopy).

### Long-Term Follow-Up

In cases of EB, 13/14 patients (93%) performed either a surveillance esophagogram (*n* = 4), an endoscopy (*n* = 3) or both (*n* = 6). A contrast esophagogram was performed in 10/14 patients (71%), showing abnormalities in 6 (60%). It was performed 10 days after ingestion in one patient to reintroduce oral feeding (normal), 3–4 weeks after ingestion in 7 patients (3 were normal, 3 had a short segment of less distensibility, and one had a mild stricture) and 2 months after the ingestion in one patient (the patient with a perforation who had the most complicated clinical evolution, in whom a long segment of severe stricture was detected). All the patients who had perforation after ingestion (*n* = 4) showed alterations: a segment with less distensibility (*n* = 2), mild stricture (*n* = 1), severe stricture (*n* = 1).

A surveillance endoscopy was also performed in 9/14 patients (64%) 3 weeks to 1 month after battery ingestion in 7 patients (which showed a mild stricture in 2, healing areas without stricture in 2, or was normal in 3), 2–3 months after in 2 patients (both normal, one of them with a mild stricture in endoscopy performed 3 weeks after ingestion), and 6–12 months after in 3 (with severe stricture requiring endoscopic dilation in 2 and mild stricture in one).

Mild stenosis (defined as a segment of lower distensibility and a diameter but without esophageal upstream dilation) was detected in 4/14 patients (29%), two of them at transition between cervical and thoracic esophagus, one at thoracic esophagus above the carina, and the last one at thoracic-abdominal esophageal transition. All of them resolved 3 months after ingestion (*n* = 3) and up to 2 years after (one patient, who still has some choking episodes while eating).

Severe stenosis requiring endoscopic dilations was observed in 2/14 patients (14%)–one had a supracarinal stenosis, which resolved after a single endoscopic dilation (although currently with some choking episodes while eating), and the other patient (who had the most complicated clinical evolution) had a long segment of stricture (10 cm) at hypopharyngeal-esophageal transition upon two unsuccessful endoscopic dilations, poor weight gain despite gastrostomy feeding, and frequent saliva-associated choking episodes. This patient has recently been submitted to an esophagocoloplasty.

In GB, surveillance endoscopy was performed in only 1/21 patients (5%, one of the patients who had a Grade IIIa gastric injury) 1 month after ingestion, which was normal.

## Discussion

Battery ingestions in children continue to rise due to a growing use of button batteries in household products (1, 2, 5, 12). In our study, we also reported a growing incidence of battery ingestions in the last 6 years. The National Poison Data System (USA) outlined 83,459 battery ingestions from 1985 to 2017, 77% in children younger than 6 years (13). A higher rate of major complications (0.8%) and mortality (0.15%) due to button battery ingestions has been reported, with a seven 7-fold increase in the relative risk of severe morbidity in the last two decades (9). This could be explained by the more frequent use of larger (20 mm) lithium button batteries (2, 7, 14). Necrosis within the esophageal lamina propria may begin as soon as in 15 min from the time of ingestion, with extension to outer muscular layers in 30 min and causing severe damage in 2 h (4, 7–9). Continued injury may occur even after removal of the battery, with development of AEF reported more than 3 weeks after removal (8). Esophageal batteries are the most problematic due to the alkaline environment, increased risk of lodgment, and close contact with the respiratory tract and major vessels (9). We also verified that in our sample, in which all the patients with EB had acute or long-term complications, a statistically significant association between esophageal location and presence of severe mucosa injury.

Damage due to battery ingestion is caused through three main mechanisms: direct pressure on the mucosa (pressure necrosis), leakage of battery contents (chemical damage), and electrical current generated by contact against the mucosa (electrical damage). This last mechanism is the primary cause of BB mucosal injury due to a rapid rise in pH caused by hydrolysis of water and, consequently, hydroxide ion generation at the negative pole (anode), causing burn and liquefactive tissue necrosis due to the creation of an alkaline environment (2, 7, 9, 15). The most rapid and severe injuries are noted with 3-V lithium batteries, although 1.5-V batteries can also cause significant injury but at a slightly slower rate. BB esophageal injury does not appear to have a thermal injury component (15). Even used batteries can retain sufficient residual voltage to cause damage (9).

Children usually take the batteries from household products. In one study (7), it is stated that 61.8% of ingested batteries in children younger than 6 years were removed from a product (mainly from hearing aids, remote controls, and toys), 29.8% were discarded batteries, and 8.2% were removed from the packaging. In our study, although we had no data on the source of the battery, we reported a high number of ingestions at home.

Button batteries > 20 mm represent a greater risk for mucosa injury, as they have a higher voltage and can easily lodge in the esophagus (2, 7, 16). Using several databases, Litovitz et al. (17) analyzed more than 60,000 cases of battery ingestions and noted that the specific battery most likely to cause serious sequelae was the ≥ 20 mm button battery, associated with 92.1% of major or fatal complications. In our study, all ingested batteries were lithium ones, and 97% were button batteries, 62% > 15 mm, in accordance with literature (13). The diameter of batteries lodged in the esophagus was similar to the size of gastric ones (batteries ≥ 15 mm were 63% and 62% of EB and GB, respectively), and no statistically significant correlation between the size of the battery and the risk of esophageal impaction nor the severity of mucosa lesions was identified.

Battery ingestions are more frequent in small children, and associated complications are also more prevalent in children <5 years (7, 9). In our study, the median age was 26 months, similar to what is reported in other series (5, 6, 18), and 75% of children were younger than 5 years. Despite perforation in our sample was more frequent in younger patients, this relationship was not statistically significant either. Unwitnessed ingestion is another well-known risk factor in complications (16). Despite almost all ingestions in our sample were witnessed ingestions (86%), all cases of unknown history of ingestion (*n* = 5, 14%) had an esophageal location and presented with acute or chronic complications. In these patients, the main reason for going to the ED was fever (100%). Most of the children in our series were previously healthy (80%), although two of them had developmental disability, another well-recognized risk factor in foreign body ingestions.

Symptoms of battery ingestion are non-specific and can be seen in other diseases such as viral infections, which may cause a delay in diagnosis if the ingestion was unwitnessed. Acute symptoms, usually related to EB impaction and mucosal injury, include vomiting, sialorrhea or stridor. Symptoms of potential complications (which usually appear a long time after ingestion) comprise fever, hematemesis, chest pain, and neck stiffness (4). In our sample, almost 60% of patients were symptomatic. The symptoms were much more frequent in EB (100 *vs*. 24% of GB), similar to another studies (19). The most frequent symptom in both EB and GB was vomiting (50%), followed by sialorrhea (30%), fever (20%), and refusal to eat and prostration (15%). These results are similar to what is reported in literature (5, 13, 19, 20) and reflect the high number of cases in whom a later than recommended removal was performed, showing symptoms of potential complications. In patients with GB, two had esophageal lesions that could explain these symptoms (one patient with abdominal pain had a Grade IIa esophageal and gastric injury, and another one with vomiting and sialorrhea also had a Grade IIa esophageal lesion).

All patients with suspected battery ingestion must perform a two-view (anterior-posterior and lateral) X-ray of the neck, chest, and abdomen to identify the “double ring” or “halo” sign, which can distinguish a battery from a coin (1, 9). In our sample, 40% had an EB and 60% a GB. Emergent removal of EB is the key to prevent complications, as the esophagus is the most frequent location of severe mucosal damage. Recent guidelines of the ESPGHAN of 2021 (9) and the NASPGHAN) of 2015 (8) have recommended to emergently remove EB (ideally in <2 h) in both symptomatic and asymptomatic children.

Management of GB remains controversial. According to some reports (9, 18, 21), complications for batteries beyond the esophagus are rare, with only 7 and 1.3% of overall complications occurring in the stomach and bowel, respectively. Additionally, according to a large Turkish study (22), a spontaneous passage through the gastrointestinal tract occurred in almost all batteries (77%) in which the battery passed the pylorus. Nevertheless, since only a few patients with GB undergo endoscopy, the frequency and the degree of gastric damage are not well-established (23). In cases of batteries beyond the esophagus, when a patient is younger than 5 years and the battery ≥ 20 mm, NASPGHAN guidelines of 2015 (8) recommend to perform an endoscopy in 24–48 h to assess esophageal injury; if the patient is older than 5 years and/or the battery < 20 mm, outpatient observation can be done, repeating the X-ray in 48 h for batteries ≥ 20 mm or in 10–14 days for those < 20 mm, if failure to pass in stool. Endoscopic removal is recommended at that time, if the battery did not pass the stomach, or at any time if symptoms appeared. On the contrary, 2021 ESPGHAN guidelines (9) recommend that, once the battery has passed the esophagus, asymptomatic patients with witnessed ingestion and in whom diagnosis of battery ingestion is performed, short time after ingestion should repeat the X-ray only after 7–14 days to confirm passage, unless the battery has been noticed in the stool. Only if it had not passed the stomach at that time, endoscopic removal would be necessary, because the chance of spontaneous passing is minimal. In cases of batteries lodged in the small bowel, surgical removal might be necessary; whereas, if located in the colon, they usually pass without intervention.

Nevertheless, the passage of the battery to the stomach does not exclude esophageal injury. Even after passage, necrosis of the esophagus and surrounding tissues progresses and can lead to severe or even fatal outcomes (1, 9). In a Korean study (18), authors performed four endoscopies to remove a GB, with a median time until removal of 5 h; all the patients had endoscopic esophageal injuries−1 major (ulcer), 3 moderate (erosions). Thus, the authors suggest urgent removal of button batteries even in cases of gastric location. In another study (24), 6/13 patients with GB performed an endoscopy. Despite the relatively early removal time (median: 27 h), mucosal gastric injury was seen in 4/6–67% (Grade I in one, Grade IIa in two, Grade IIIa also in one). The patient with the most severe mucosal injury had the GB removed in just 10 h, while a child with no mucosal lesions had the battery in the stomach for 4 days. Due to occurrence of gastric lesions in the first hours after ingestion, the authors also recommend the extraction of a GB in the first 24 h. In another series (25), 12 GBs were removed endoscopically (median time of 4 h), and mucosal injury was present in 6/12–50% (one patient had erythema, 4 erosions, one necrosis), but no complications were detected. The patients with gastric lesions tended to be younger, although this relationship was not statistically significant. The only patient with severe injury (necrosis) had the GB removed just 10 h after ingestion, so the authors also advocate for extraction of GB within 24 h. A multicentric study published in 2021 (26), the largest series of GB published to date, reported 68 patients with GB submitted to endoscopic removal, with a median age of 2.5 years (8 m−16 years) and exposure time of 9 h (3–117 h); 60% had gastric damage (ulcerations, necrosis, abrasions, erythema, friability, and erosions). One patient had gastric perforation with pneumoperitoneum (exposure time of 117 h). In this study, the patients who had a GB removed after 12 h were 4.5 times more likely to have gastric damage than those whose battery was removed before 12 h; therefore, prompt removal of GB (in the first 12 h) is advisable, according to the authors. On the contrary, other studies advocate for a more conservative approach. In an American study (19), 56/67 (84%) of GB were managed conservatively, 11 (16%) performed an endoscopy with a median time to removal of 1.5 days (5 due to symptoms, the remaining due to co-ingestion of a magnet, ingestion of more than one battery or were removed prophylactically); only 4 (36%) had erythema of the mucosa, and none of them had major complications. In the same study, out of 43 duodenal batteries, 38 (88%) were managed through a conservative approach; only two patients performed endoscopies (5%), which were normal, although three patients needed surgical intervention due to battery failure to pass. In our study, we had 3 patients with GB on the X-ray who had an esophageal injury (two had a Grade IIa injury, and one had a Grade IIIa), although none of them had acute or chronic complications. Because mucosa damage can be more severe in cases with GB and later removal, ESPGHAN guidelines (9) recommend performing an emergency endoscopy to exclude esophageal damage in specific circumstances of unwitnessed ingestion, delayed (>12 h) diagnosis in symptomatic children or if co-ingestion of a magnet.

Our strategy consists of performing an emergent endoscopy (ideally in the first 2 h) in cases of EB. Batteries in the stomach are removed as soon as possible in symptomatic children, in cases of ingestion of more than one battery and if co-ingestion with a magnet. In cases of asymptomatic children, endoscopic removal is performed if it remains in the same position after 24 h, with a follow-up X-ray to confirm passage and in cases of unknown time of ingestion. Nevertheless, as already mentioned, ultimate decision about the best timing for battery removal relies on the endoscopy team, considering each case circumstance. Larger prospective studies are needed to assess and stratify the risk for GB.

Endoscopy was effective in removing the battery in 94% of cases, a similar rate to what is described in literature (27). In 2 cases (6%), rigid esophagoscopy was necessary to remove the EB after endoscopy failure; in both cases, the batteries were lodged in the cervical esophagus. Rigid esophagoscopy performed by an otolaryngologist is reserved to EB located in the upper third of the esophagus in which endoscopic removal was not successful. This contrasts with some reports in which EBs are mainly removed by rigid esophagoscopy (19). In a recent Dutch report (28), 12.5% of batteries were surgically removed; on the contrary, we had no cases requiring surgical removal.

Late battery removal is another recognizably risk factor in complications (16). Although endoscopy was performed shortly after hospital admission in our sample, median time to battery removal in cases of EB was 7 h. Removal was performed later than recommended (≥ 6 h) in 80% of cases, as reported in other studies [with a median time from ingestion to removal of 6 h (5, 25)/7.5 h (29) and 8 h (30)] and shorter than other case reports [with 17 h (6) and 36 h (31)]. This later removal was due to late referral in 55% of cases, as most of the patients (89%) were referred from other centers; some of them remote and due to unknown history of ingestion in 45% (all of them with EB), as the patients went to the ED only after unspecific symptoms appeared. In these cases, EB removal was performed as late as 7 days after the ingestion. We emphasize a particular case, in which the delay was due to both an unwitnessed ingestion and misdiagnosis; the battery was mistaken for an artifact on the first evaluation at the ED from a secondary hospital, leading to endoscopic removal 21 days after the initial visit to the ED.

All the patients with EB had severe esophageal caustic injury, in accordance with other series (6, 13, 25, 31, 32). In our study, Grade III injury was seen in 12 (86%, Grade IIIa in 7/50% and IIIb in 5/36%) and Grade IV in two (14%). Even the patient with an EB who expelled the foreign body before endoscopy had a severe esophageal lesion (Grade IIIa), which reinforces the importance of performing an endoscopy even in these situations (in cases of EB on the X-ray). We found no statistically significant association between duration of exposure and severity of esophageal lesions, in accordance with what is reported in recent studies (6, 29). Nevertheless, we had 4 cases of perforation (29%), two confirmed by CT-scan, with a median exposure time of 2 h-8 h-7 days−7 days. A perforation rate was also similar to what was reported in other studies [Shaffer et al. (30) reported a 25% perforation rate and Panella et al. (31) a 33%]. In a larger study of 290 cases concerning battery ingestions in USA with severe or fatal outcomes (20), the shortest time from ingestion to perforation was 18 h, and the perforation risk was only 2% on the 1st day, 9% on the first 48 h and 27, 37, 46, and 66% on the 3rd, 4th, 5th, and 9th days, respectively; thus, safe time until perforation was considered to be 11–12 h. In another study, although the perforation rate was similar, it was rare in the first 12 h (9)^.^ These results contrast with ours–with perforation detected as soon as in 2 h after ingestion.

Even though in patients with GB some degree of mucosal damage was seen in 70%, almost all lesions were minor injuries (93%); only one patient had a major lesion (Grade IIIa esophageal ulceration), an asymptomatic 6-year-old patient with a witnessed ingestion of a 20-mm button battery in whom endoscopic removal was performed 16 h after ingestion. In another two patients, an esophageal lesion was identified (both Grade IIa), which is in accordance with the reported evidence, showing that the passage of the battery to the stomach does not exclude esophageal injury, especially if the time until passage to the stomach is unknown. Endoscopic removal in patients with GB should be weighed, not only to identify mucosa gastric damage (as it is usually mild in most cases), but to rule out potential serious esophageal lesions.

Some mitigation strategies have been proposed to diminish the chance of esophageal injury. Battery removal may be performed later than recommended due to late referral to a center with availability of pediatric endoscopy. In such cases, early ingestion of honey and/or sucralfate in the clinical setting may help slow the rate of esophageal injury until the BB can be removed and, thus, improve patient outcomes (9, 33, 34). In the work by Anfang et al. (33), sedated piglets were randomized to receive 10 ml of honey, sucralfate, or saline 10 min post button battery placement in the esophagus and every 10 min thereafter. The honey and sucralfate neutralized esophageal tissue and reduced esophageal burns. Half of the saline control group developed esophageal perforations 1 week after ingestion compared with no perforations in the piglets treated with honey or sucralfate. In accordance with these data, the National Capital Poison Center (NCPC) recommended its use in the first 12 h after suspected or witnessed ingestions (35). The mechanism of action is thought to be limiting electrolysis by coating of the battery and neutralization of generated hydroxide, as both honey and sucralfate are weak acids (9). Although esophageal perforation is less likely in the first 12 h after ingestion, this period does contain the peak of electrolysis activity and battery damage. Therefore, giving honey and/or sucralfate might be considered in ingestions ≤ 12 h while waiting for endoscopic removal but should not delay it (9, 34). Parents calling the emergency room may be advised to directly start giving honey if the history is strongly suggestive of BB ingestion, and no signs of perforation are present (9). The advised dose for both is 10 ml every 10 min with a maximum of 6 doses of honey over the age of 12 months and 3 doses of sucralfate, respectively (34).

Nevertheless, these data are based on *in vitro* and *in vivo* studies of piglets, while human studies are still lacking. Additionally, we must be cautious in cases of delay in diagnosis, clinical suspicion of perforation, mediastinitis, sepsis, swallowing difficulties, allergies to honey or sucralfate, and in children <1 year of age because of the risk for infant botulism with honey intake (9).

Regarding post removal mitigation strategies, Anfang et al. (33) suggested that irrigation of tissue with 50–150 ml of 0.25% sterile acetic acid after battery removal can help neutralize the highly alkaline substrate in an effort to reduce progression of injury after BB removal. This strategy was applied in the 6 pediatric patients' series of Jatana et al. (15). All cases were reported to have improved mucosal appearance after irrigation, and none of the patients experienced perforation or stricture formation. This is thought to be because of immediate pH neutralization toward a physiologic range and arrest of liquefactive necrosis.

Mitigation strategies are not done at our center, but we will consider implementing pre-removal mitigations strategies in the near future. More evidence is needed to consistently suggest the benefit of post-removal strategies.

Performing a CT-scan before endoscopy is recommended in cases of prolonged ingestion or signs of perforation and after endoscopy in cases of major endoscopic lesions to exclude perforation and vascular injury (4, 9). In our study, CT-scan was performed in 9 patients (26%), all of them with EB, in 4 cases before endoscopy and in 5 cases after it, confirming perforation in 3 cases (two of them not detected endoscopically).

Short-term evolution was much worse in patients with EB than in those with GB, as expected, although the PICU admission rate was lower in our sample than in other studies (1). All the patients (14/14) with EB were hospitalized, and 4 (29%) admitted to the PICU, with a median length of stay of 21 days. Only 2/21 patients with GB (10%) were admitted to the pediatric ward, none of them in the PICU, and the length of stay was 9 h. The complications after a battery ingestion described in literature include vascular injury and bleeding events, esophageal perforations, vocal cord palsy, pneumothorax, aspiration pneumonia, mediastinitis, spondylodiscitis, TEF, AEF (that can present up to 4 weeks post-removal), and strictures, which may take months to occur (5, 7–9). In our series, the overall rate of complications was infection, 20%; perforation, 11%; pneumomediastinum; RDS and stridor, 6%; and pneumothorax, subglottic stenosis, and hemodynamic instability, 3%. More than half of the patients with EB (57%) presented with acute complications, but no acute complications were detected in patients with GB.

Complication rates in EB are heterogenous, depending on the study nature. In the 20-year (1995–2015) systematic review of Varga et al. (36), 226/136.191 children (0.16%) had complications after button battery ingestion, including perforation, 18%; stricture, 14%; TEF, 15%; vascular involvement, 6%; bilateral cord palsy, 2%; pneumonia,0.4%; spondylodiscitis,0.4%. The 61 fatal outcomes (0.04%) should be emphasized, −27 (44%) due to AEF or other fistula formation, 11% due to suffocation secondary to blood aspiration/pneumonia, and 44% of an unknown cause.

In a recently published Indian study (29), only 5/52 children had complications (10%), all of them with Grade III caustic injury and 80% with unwitnessed ingestion. The complications were pneumothorax in 4%, and pneumomediastinum, TEF, and AEF each one in 2%. Lahmar et al. (6) reported a 27% complication rate (pneumomediastinum in 8%, and mediastinitis and vocal cord palsy in 4% each), similar to Panella et al. (31) (the complication rate of 33%–TEF and perforation in 17% each). In the multicenter American study of Shaffer et al. (30), perforation was observed in 25%, TEF in 8%, sepsis in 6%, mediastinitis, vocal cord palsy, and osteomyelitis in 4% each and pneumomediastinum and spondylodiscitis in 2% each.

Although our overall complication rate was higher than reported in most of the studies, we did not observe any case of TEF, AEF, or osteoarticular infections, neither any mortality case. This contrasts with most series, in which the rate of TEF varies between 2 and 8% (13, 29, 30) to more than 10% (5, 31) and AEF between 1 and 17% (29, 32) and mortality between 2 and 17% (1, 5, 13, 28, 29, 32) up to 25% (27), mostly due to hemorrhagic or septic shock in the context of AEF or TEF. In accordance with literature, in our series, no patient with GB developed acute complications. In the American study previously reported (30), they had 6 cases of GB, 5 of them removed with both endoscopy (80%) and open surgery (20%). Time to removal was 14.5 h (4–96 h); gastric ulceration was detected in 33.3% and Grade I esophagitis in 16.7%. Feeding support was required in 34% of all our patients–in 79% of EB and in 5% of the patients with GB (enteral nutrition), and, similar to other studies (6, 13), one patient with an EB needed a gastrostomy placement (7% of EB, 3% of all batteries). In the same American study (30), feeding support was needed in 56.3% of patients with EB and in 16.7% of patients with GB.

Contrast esophagograms and/or repeated endoscopies are necessary to detect stricture formation. A second look endoscopy, 2–4 days after ingestion could be important to determine the timing of feeding introduction, but this may lead to false reassurance about continued risks for complications; AEF can only present 3 weeks after, and stricture does not often present before 4 weeks after ingestion (1). Follow-up care is essential to assess for mid and long-term sequelae (5). Early dilation of a stricture will lead to a better swallowing function; however, the procedure should be delayed until 4 weeks post ingestion for the tissue to heal and reduce the risk of iatrogenic perforation (4, 9).

Concerning chronic complications, stenosis was present in 6/14 of our patients with EB (43% of EB, 17% of all patients) but only severe (submitted to endoscopic dilations) in 2 (14% of EB, 6% of all patients). Reported stricture rates are also diverse–in different studies, ranging from 8 to 17% (6, 32) to as high as 42% (5). In all our patients with mild stenosis (4 cases, 29%), it resolved without dilation. In the 2 patients with severe stenosis, one resolved after a single endoscopic dilation and the other presented failure to endoscopic dilation and required an esophagocoloplasty. Median follow-up time of EB in our sample was 2 months; 4 patients maintain longer follow-up: 10 months, 2 and 10 years after ingestion (due to choking episodes, although none of them had dysphagia and had a normal endoscopy) and one patient after 3 years due to poor weight gain despite gastrostomy feeding (patients submitted to an esophagocoloplasty). In an Australian study, more than half of a cohort of 51 children revealed dysphagia at the time of the discharge (4). In an Indian study (29), although follow-up esophagogram was normal in all patients (50/50), occasional symptoms like cough (18%) and mild chest pain at swallowing (10%) were observed but resolved.

Because battery ingestion remains a relevant public issue due to its high-associated morbidity, some efforts have been made to diminish this burden through primary prevention and product innovation and redesign (2, 7, 12). One of the recent advances in this area has been made by Landsdowne Labs^®^, a battery technology company that has created a new mechanism to deactivate coin cell batteries upon ingestion, limiting severe mucosal injury. Also, stricter legislation for screw-secured battery compartments in devices and retail packing is essential (5). Both BB and electronics manufacturers should also consider instructing the use of common household tape options to cover BB immediately after removal from a device for either recycling or disposal. Such precautions may help to reduce related ingestion injuries in children, as shown in a recent study, in which BBs were wrapped with different types of common household tapes; none of the tape-wrapped batteries showed voltage changes nor presented any hazard stemming from BB ingestion. Additionally, the 6-covered batteries placed in the cadaveric piglet esophageal tissue model demonstrated no visible tissue injury and no change in tissue pH in contrast to the controls (37).

As an effort to increase awareness about battery ingestion and due to the lack of reporting of many cases, an anonymous reporting of BB injuries can be made through the advent of a smartphone application called the Global Injury Research Collaborative application (“GIRC app” ^®^); it can be downloaded from both the App Store (iOS) and Google Play (Android) for no charge by medical professionals. It is a deidentified, anonymous, efficient worldwide smartphone app database for all aerodigestive foreign body cases for purposes of documenting events, measurements, and photographs of the foreign body removed and clinical outcomes (38).

### Study Strengths and Limitations

Despite concerning a single-center study, our study included a relatively large series of patients, as compared to other similar studies at the same time period and considering its regional and temporal representativity. Furthermore, our data also represent the most recent experience of a dedicated emergency endoscopy team, providing specialized care and support to the Lisbon area and to the southern part of the country. To the best of our knowledge, this is the first reported series concerning a Portuguese pediatric population. Nevertheless, studies with a higher sample size will be necessary to find a more accurate relationship between duration of impaction and clinical outcomes.

Although some information was missing or not registered in patient's electronic clinical files, a quite detailed clinical description associated with battery ingestion, with a focus on a clinical profile, mucosal lesions and short- and long-term complications, was performed. The availability of actual and detailed data from different national centers with expertise and specialized resources in managing battery ingestion based on their respective experience represents a valid contribution to the establishment of networking and to support advocacy efforts among stakeholders, including industry representatives and policy makers, in preventing worldwide button battery injury.

## Conclusion

Despite ongoing joint efforts of professionals and scientific societies, button battery ingestion remains a worldwide relevant public issue, and specific expertise is required in managing its complications. At our setting, referral due to battery ingestion has recently increased, in parallel with associated severe complications, despite the availability of an emergency pediatric endoscopy team. As expected, the patients with EB had more severe mucosal injuries and more complicated short and long-term outcomes. Although the patients with GB had milder lesions, the presence of a GB could not exclude esophageal injury. Taking into account the critical time, devastating impact, as well as the challenges of identifying and managing button battery ingestion, primary prevention remains critical, in parallel with injury mitigation strategies through product innovation and redesign.

Parents and child caregivers must be informed about the hazard and appropriate actions to take to prevent ingestions. Beyond prevention, early recognition and removal of the battery in referral centers with specific expertise represent the next level of defense against long-term complications.

## Data Availability Statement

The raw data supporting the conclusions of this article will be made available by the authors, without undue reservation.

## Ethics Statement

Written informed consent was obtained from the individual(s), and minor(s)' legal guardian/next of kin, for the publication of any potentially identifiable images or data included in this article.

## Author Contributions

CL was responsible for the acquisition and analysis of patient's data, carried out bibliographical research, and drafted the initial manuscript. CL and AL planned and conducted the design of the present study. SA, AF, HL, PM, and AL performed all endoscopies. SA contributed to writing the manuscript. SA, AF, HL, and PM revised and approved the manuscript. AL supervised and approved the final version of the manuscript. All authors contributed to the article and approved the submitted version.

## Conflict of Interest

The authors declare that the research was conducted in the absence of any commercial or financial relationships that could be construed as a potential conflict of interest.

## Publisher's Note

All claims expressed in this article are solely those of the authors and do not necessarily represent those of their affiliated organizations, or those of the publisher, the editors and the reviewers. Any product that may be evaluated in this article, or claim that may be made by its manufacturer, is not guaranteed or endorsed by the publisher.

## Abbreviations

AEF: aortoesophageal fistula
CT: computed tomography
CVC: central venous catheter
ED: emergency department
EB: esophageal battery
GB: gastric battery
PICU: pediatric intensive care unit
PPI: proton pump inhibitor
RDS: respiratory distress syndrome
TEF: tracheoesophageal fistula.
